# Comment on "Predictive efficacy of immature granulocytes in acute complicated appendicitis"

**DOI:** 10.1590/1806-9282.20250286

**Published:** 2025-07-07

**Authors:** Enes Ağırman, Rıfat Peksöz, Sabri Selçuk Atamanalp

**Affiliations:** 1 Erzurum City Hospital, Department of General Surgery – Erzurum, Turkey.; 2 Atatürk University Research Hospital, Department of General Surgery – Erzurum, Turkey.

Dear Editor,

We have read with great interest the valuable article written by Yazla et al., titled "Predictive efficacy of immature granulocytes in acute complicated appendicitis." The authors aimed to provide evidence on whether immature granulocytes (IG) and other laboratory markers could serve as useful tools in the diagnostic workup of appendicitis, particularly in distinguishing between complicated and uncomplicated cases in this study^1^.

Acute appendicitis is the most common surgical emergency. Laboratory values that can help diagnose the disease and determine whether the appendix has become complicated are of great importance. The ability to diagnose the disease using only clinical findings and laboratory values, without the need for advanced radiological imaging methods such as ultrasonography and computed tomography, is a significant advantage. This is particularly important for physicians in rural areas and primary care settings who may have limited access to imaging methods. Laboratory tests such as hemogram and biochemistry are inexpensive, are easily accessible, and provide quick results. Therefore, this study, which emphasizes the importance of laboratory values in diagnosing acute appendicitis and determining its complicated status, is valuable and will make a significant contribution to the literature. Additionally, the discussion of a diagnostic marker such as IG, which is not routinely used, further highlights the importance of the study. However, we believe that reevaluating and discussing certain aspects of the authors’ study will contribute to the current study and future studies. We would like to share some comments on the study.

First, the study discusses the effectiveness of blood ­values in determining complicated appendicitis. However, the inclusion of a control group could make the study even more ­meaningful. This would allow for a discussion of the importance of laboratory values in diagnosing acute appendicitis as well. The control group could consist of healthy controls, a negative appendectomy group, or patients hospitalized for abdominal pain and treated medically. We believe that dividing patients into groups (control group [healthy controls or negative appendectomy group], uncomplicated group, and complicated group) would be more valuable^2,3^.

Second, the article identifies IG percentage as a useful predictive biomarker with 92% specificity for determining complicated appendicitis. While the specificity of IG is indeed very good, its sensitivity is very low (23%). When evaluating diagnostic efficacy, it is important to consider both sensitivity and specificity, as well as the area under the curve (AUC). A high specificity alone is not sufficient. In our opinion, the diagnostic superiority of neutrophil and neutrophil-to-lymphocyte ratio (NLR) values in the authors’ study is better than that of IG.

Third, the study does not include mean platelet volume (MPV), which is an important hemogram sub-parameter. In recent years, MPV has become frequently used in the diagnosis of inflammatory diseases^3^. In the study by Albayrak et al. on acute appendicitis, MPV was found to have a high diagnostic value. This article has been referenced in numerous studies^4^. In our study, MPV was also found to be a significant hemogram sub-parameter in diagnosing acute appendicitis and indicating the likelihood of complications^3^. Therefore, we recommend that the authors analyze MPV values, as this parameter may prove more significant compared to other markers. Alternatively, if this parameter has already been studied, we are curious about the results.

Lastly, to better understand the clinical characteristics of the patient groups and to compare the groups based on these ­characteristics, Table 1 should include parameters such as length of hospital stay and postoperative complications. Additionally, in Table 2, presenting the C-reactive protein (CRP) value in mg/dL would improve the readability of the table.

We congratulate the authors for their successful study and await their responses to our comments.

## Data Availability

The datasets generated and/or analyzed during the current study are available from the corresponding author upon reasonable request.
